# Robust Performance of the Novel Research-Use-Only Idylla GeneFusion Assay Using a Diverse Set of Pathological Samples with a Proposed 1-Day Workflow for Advanced NSCLC Evaluation

**DOI:** 10.3390/cancers15010292

**Published:** 2022-12-31

**Authors:** Alvaro Leone, Lucia Anna Muscarella, Paolo Graziano, Andrea Tornese, Lucia Rosalba Grillo, Angela Di Lorenzo, Monica Bronzini, Stefania Scarpino, Angelo Sparaneo, Giulio Rossi

**Affiliations:** 1 Anatomic Pathology Unit, San Camillo-Forlanini Hospitals, 00152 Rome, Italy; 2 Laboratory of Oncology, Fondazione IRCCS Casa Sollievo della Sofferenza, San Giovanni Rotondo, 71013 Foggia, Italy; 3 Unit of Pathology, Fondazione IRCCS Casa Sollievo della Sofferenza, San Giovanni Rotondo, 71013 Foggia, Italy; 4 Department of Clinical and Molecular Medicine, Pathology Unit, St. Andrea University Hospital, University of Rome La Sapienza, 00187 Rome, Italy; 5 Operative Unit of Pathologic Anatomy, Department of Oncology, Fondazione Poliambulanza Hospital Institute, 25124 Brescia, Italy

**Keywords:** non-small-cell lung cancer, ALK, ROS1, RET, MET, NTRK

## Abstract

**Simple Summary:**

Updated international guidelines suggest NGS as the preferred procedure for NSCLC patients’ evaluation for predictive biomarkers, but NGS facilities are not available everywhere. Alternative molecular techniques are rapidly evolving, each with different characteristics and performance in terms of turn-around time, sensitivity, specificity and required personnel skills. Among these, the novel Idylla^TM^ GeneFusion assay, a rapid and fully automated platform able to simultaneously detect *ALK*, *ROS1*, *RET* and *NTRK1/2/3* and *MET* ex14 skipping mutations, is emerging and deserves validation as a stand-alone test for laboratories lacking NGS facilities or as an alternative option for ultra-rapid NSCLC patient profiling when time efficient NGS evaluation is not possible.

**Abstract:**

A range of different techniques are available for predictive biomarker testing for non-small-cell lung cancer (NSCLC) clinical management. International guidelines suggest next-generation sequencing (NGS) as the preferred procedure, but other reverse transcriptase-polymerase chain reaction (RT-PCR)-based methods are rapidly evolving. In this study, we evaluated the reliability and accuracy of the Idylla^TM^ GeneFusion assay, a rapid and fully automated platform able to simultaneously detect *ALK*, *ROS1*, *RET* and *NTRK1/2/3* and *MET* ex14 skipping mutations and compared its performance with routine reference methods. The cohort included thirty-seven NSCLCs plus two parotid gland carcinomas, previously characterized for the above alterations through either IHC, FISH, RT-PCR or NGS. In 36 of 39 cases, the Idylla GeneFusion assay and the reference methods were concordant (overall agreement: 92.3%). Tumor sections stored at room temperature for up to 60 days and 17 cases older than 2 years were successfully characterized. Our results suggest that the Idylla GeneFusion assay is a reliable tool to define gene fusion status and may be a valuable stand-alone diagnostic test when time efficiency is needed or NGS is not feasible.

## 1. Introduction

In recent years, the treatment of patients with advanced NSCLC has dramatically improved due to the identification of specific targetable oncogenic driver mutations that have fueled the development of novel therapeutic strategies. Since the pioneering discovery of *EGFR* mutations in 2004, several other oncogenic driver alterations have been identified, leading to the development of a rich armamentarium of effective targeted agents and, consequently, to unprecedented progress in the treatment of oncogene-dependent NSCLC [1,2,3].

This decline in mortality from NSCLC has been mainly attributed to an improvement in survival due to these new treatment options that are superior to conventional chemotherapy [4]. The incidence and type of oncogene mutations depend on clinical variables (e.g., sex, race, smoking habits) and tumor-associated factors such as histology and stage. In non-squamous carcinoma histology, the most frequently detected alterations in Caucasian individuals are gene mutations involving *KRAS*, *EGFR*, *BRAF*, *HER2*, plus *ALK*, *ROS1*, *RET* and *NTRK1/2/3* rearrangements and *MET* exon 14 skipping mutations. For all these molecular alterations, there are specific drugs that have shown promising activity in several clinical trials [5]. A wide array of different testing modalities can be used for detecting gene fusions/rearrangements at the DNA, RNA or protein level. The most widely used methods so far have been based on the detection of the expression of the relative fusion protein via immunohistochemistry (IHC) or at the genomic DNA level via fluorescent in situ hybridization (FISH). Both methods are applicable to histological and cytological samples with a low tumor cell content. IHC for the ALK and ROS1 proteins and, more recently, for NTRK1/2/3, is routinely used to detect the corresponding gene rearrangements in most pathology laboratories [6,7,8,9]. However, the continuous increase in the number and complexity of the events to be deciphered makes a single-test approach uneconomic and time-consuming. Therefore, the routine implementation of new, robust and comprehensive multitesting methods is eagerly needed. In this regard, the NGS technology is gaining more and more appreciation thanks to its characteristics of sensitivity and rapidity [10,11,12,13].

On the other hand, as an alternative to NGS, RT-PCR multitests capable of simultaneously detecting multiple gene rearrangements in a fast and robust way are also emerging [14,15,16]. The novel research uses only the (RUO) Idylla^TM^ GeneFusion assay (Biocartis), performed on the Idylla^TM^ Platform, and consists of a fully integrated and automated cartridge-based assay providing deparaffinization and digestion of the tissue up to mRNA amplification via real-time-PCR. The assay may simultaneously detect the presence of fusions involving ALK, ROS1, RET and NTRK1-3 and MET exon 14 skipping in about 3 h, although recognition of different gene partners cannot be performed. Moreover, 3′/5′ EI assays have the advantage that information on the presence of a fusion may be provided when fusion transcripts with uncommon breakpoints or unknown fusion partners occur.

In this pilot study, the performance of the Idylla^TM^ GeneFusion Assay was assessed on a panel of thirty-seven NSCLCs and two parotid gland carcinomas, previously investigated with different testing modalities for *ALK*, *ROS1*, *RET* and *NTRK1/2/3* translocations and the *MET* ex14 skipping mutation.

## 2. Materials and Methods

The study cohort consisted of 37 NSCLC cases previously screened for *ALK*, *ROS1* and *RET* fusions and *MET* ex14 skipping, plus 2 parotid gland secretory carcinomas positive for *NTRK* gene fusion, all assessed using FFPE pathological specimens. All NSCLC cases were classified according to the fifth edition of the World Health Organization [17]. Reference methods for gene fusion detection included IHC, FISH, RT-PCR and NGS. For this pilot study, we chose surgical specimens, biopsies or cell blocks with >30% tumor cell content, as evaluated on archival hematoxylin and eosin-stained (H&E) slides. All immunostains were performed using an automated immunostainer platform (Benchmark ULTRA, Ventana/Roche, Tucson, AZ, USA) and the following antibodies: ALK (clone D5F3, companion diagnostic Ventana/Roche), NTRK (clone pan-TRK EPR17341, companion diagnostic Ventana/Roche) and ROS1 (clone SP384, companion diagnostic Ventana/Roche). FISH for *ALK* (Vysis Abbott dual color break apart, Abbott Park, IL) and *ROS1* (Vysis cytotest break apart) was performed using an Abbott VP200 processor for pre-treatment. RT-PCR for *RET* gene fusions or *MET* ex14 skipping was performed using either EasyPGX^®^ ready ALK, ROS1, RET, MET (Diatech Pharmacogenetics, Jesi, Italy), AmoyDx^®^ PLC Panel (AmoyDx, Xiamen, China), or Oncomine Dx Target Test (Thermo Fisher Scientific, Waltham, MA) for NGS analysis. The samples, specifically prepared for this purpose, were reassessed using the Idylla^TM^ GeneFusion assay (Biocartis NV, Mechelen, Belgium). Sequential freshly prepared unstained 4 µm thick sections were used, the last of which was H&E stained to re-evaluate tumor cell content after slide preparation. Pathologic sections from 1 to 5 were used for the Idylla gene fusion evaluation, depending on specimen dimension, and following manufacturer’s instructions. The paraffin tissue was dissected from the slides, inserted into RNase on wetted Whatman paper circles, and placed into the cartridge in agreement with the manufacturer’s instructions.

The study was conducted in accordance with the precepts of the Helsinki Declaration; all data were handled anonymously and in accordance with local institutional ethical board protocols in accordance with the criteria of “The Italian Data Protection Authority” (http://www.garanteprivacy.it/web/guest/home/docweb/-/docwebdisplay/export/2485392; access date: 1 September 2022).

## 3. Results

### 3.1. ALK Evaluation

#### 3.1.1. ALK Cohort

Twenty-nine total determinations with the Idylla^TM^ GeneFusion assay were performed on pathological materials of different types relative to twenty-two individuals (thirteen males/nine females; mean age 61.1 years, range 41–87) all affected by NSCLC with adenocarcinoma histology except one with large cell neuroendocrine carcinoma and one with combined small cell carcinoma and adenocarcinoma (Table 1). The pathological materials analyzed consisted of five surgical specimens, fourteen bronchial biopsies, four pleural biopsies and three lymph nodes from surgical resections. In five of the above cases, more than one determination was made using a different specimen or different pre-analytical conditions.

Twenty of these cases had previously been analyzed for ALK using IHC, one analyzed via FISH and one assessed elsewhere via RT-PCR using the AmoyDx^®^ PLC Panel (AmoyDx, Xiamen, 361027, China) technology within a clinical study. The year of creation of the paraffin blocks ranged between 2012 and 2022. Eighteen of the twenty cases tested via IHC were frankly positive, presenting the membrane staining characteristics resulting from the overexpression of a rearranged ALK protein (100% positive neoplastic cells), while two exhibited moderate and patchy staining in 50% of neoplastic cells.

#### 3.1.2. ALK Detection Workflow

**Figure 1:**
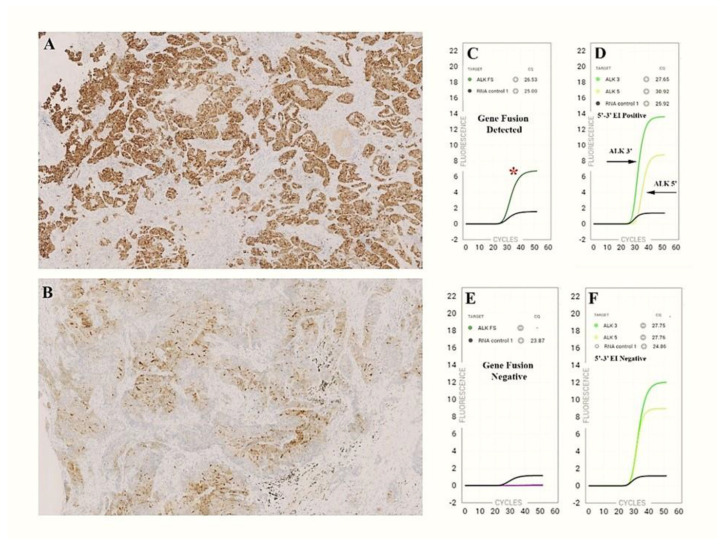
The Idylla^TM^ GeneFusion assay in comparison to IHC staining. (**A**) ADC presenting the membrane staining characteristics resulting from the overexpression of a rearranged ALK protein (100% positive neoplastic cells); (**B**) LCNE carcinoma exhibiting moderate and patchy staining in 50% of neoplastic cells; (**C**,**D**) Idylla^TM^ GeneFusion plots showing *ALK* fusion-specific amplicon (red asterisk) and 3′/5′EI (green curves); (**E**,**F**) the ALK-FS probes failed to amplify and 3′/5′ EI is not present.

Cases #1, 2, 3, 4 and 5 were analyzed within 24 h of section preparation on glass slides. In four of the first five cases analyzed, the Idylla^TM^ GeneFusion test was positive, in agreement with positive IHC ALK results. Case #4, with large cell neuroendocrine carcinoma histology, exhibited a mild and peculiar reactivity not characteristic of the conventional expression pattern resulting from ALK rearrangement. The evaluation via Idylla was negative (Figure 1). FISH analysis performed later was also negative. To verify the adequacy of sections prepared and stored for later analysis, case #5 was repeated 21 days after the preparation of the slides, which were kept in the dark in a slide box at room temperature (RT).

The test replication confirmed the previous positive result, also exhibiting overlapping Cqs of the RNA and DNA controls (cases #5 and #5-B) (Table 1). The remaining 17 cases of the ALK cohort were then analyzed at variable times between 2 and 7 days from slide preparation. Sixteen of the seventeen showed concordance between IHC and the Idylla^TM^ GeneFusion test. Case #14 related to a combined carcinoma with dubious ALK expression was negative with both the Idylla and FISH approaches. To further test the validity of the Idylla assay, case #1 and cases #15–18 were reanalyzed using different pathological specimens from the same individuals.

Case #1 on a bronchial rebiopsy after disease recurrence (#1-B), cases #15 and #16 on lymph node metastases (#15-B and #16-B) and case #18 on pleural metastasis (#18-B), while case #17, relating to a surgical specimen from 2012, was repeated on a concomitant lymph node metastasis (#17-B) and a subsequent bronchial rebiopsy performed about 10 years later during a follow up (#17-C) (Table 1). In all instances, the test was found to be concordant. In total, excluding the two cases with unclear ALK expression, the concordance between the Idylla^TM^ GeneFusion test and ALK IHC was 100%.

### 3.2. ROS1/RET/NTRK/MET ex14 Skipping Evaluation

#### 3.2.1. ROS1/RET/NTRK/MET ex14 Skipping Cohort

Twenty-six total determinations with the Idylla^TM^ GeneFusion test were carried out on pathological materials of different types relative to seventeen individuals (seven males/ten females; mean age 64 years, range 31–81) all affected by NSCLC with adenocarcinoma histology, except two with secretory carcinoma of the parotid gland (Table 2). Seven surgical specimens, five bronchial biopsies, four pleural biopsies, three cytology specimens and one lymph node metastasis specimen were included.

In seven of the above cases, more than one determination was made using different pathological materials or different pre-analytical conditions. Seven of the nine ROS1-positive cases had been previously analyzed for ROS1 in our laboratory through FISH and two elsewhere with IHC. The three RET, the two NTRK and the three MET ex14 skipping specimens had been tested elsewhere with either RT-PCR, NGS or IHC (Table 2). The date of collection of the paraffin blocks was between the years 2015 and 2022.

#### 3.2.2. ROS1/RET/NTRK/MET ex14 Skipping Workflow

The first analysis of the 17 cases was performed within 7 days of slide preparation.

**Figure 2:**
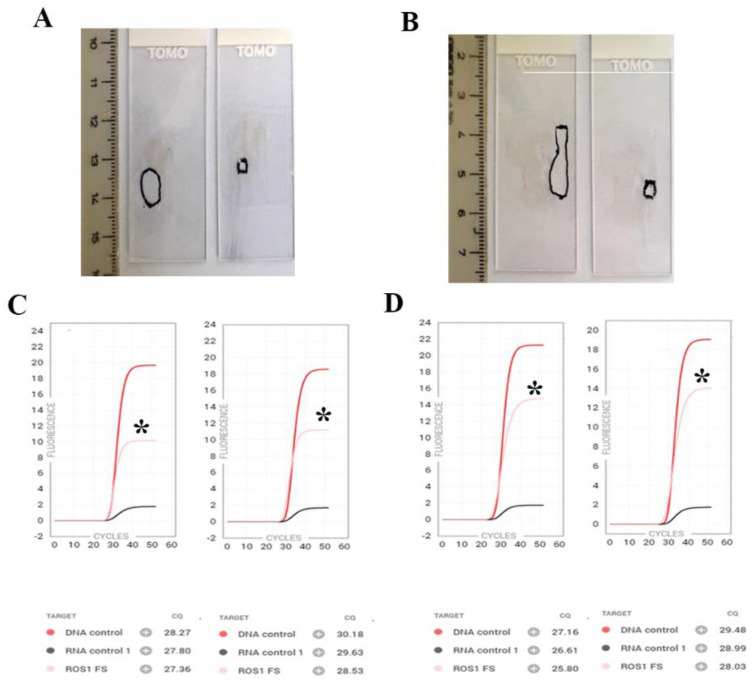
Reproducibility of the Idylla^TM^ GeneFusion assay using tumor areas of different sizes. (**A**,**B**): larger and smaller microdissections from sequential sections of ROS1-positive cases #27 (**A**) and #28 (**B**); (**C**,**D**): corresponding Idylla GeneFusion plots showing *ROS1* fusion-specific amplicons (black asterisks) in both specimens. Red curve: DNA control; black curve: RNA control; pink curve: *ROS1* fusion-specific amplicon. The surface of microdissected area in panel (**A**): was 54 squared mm on the left and 9 squared mm on the right; in panel (**B**), the microdissected surface was 70 squared mm on the left and 12 squared mm on the right.

In 14 of 17 cases, the Idylla^TM^ GeneFusion assay was concordant with the reference method. Two *ROS1*-positive FISH cases (#25 and #26) were not identified through Idylla. Case #34, relating to an adenocarcinoma, tested positive with NGS for the *MET* ex14 skipping mutation and was negative with Idylla. A third RT-PCR-based test, the EasyPGX^®^ ready *ALK*, *ROS1*, *RET*, *MET* (Diatech Pharmacogenetics), confirmed the negative result, in agreement with Idylla. To verify the analytical sensitivity of the Idylla assay, cases #27 and #28 (*ROS1* positive) were reanalyzed on sequential sections, performing a second microdissection aimed at obtaining a smaller pathological specimen mimicking a small biopsy (Figure 2). Reanalysis results were concordant, albeit with a relative increase in Cq of DNA and RNA of approximately two cycles (cases #27 and #27-B, #28 and #28-B) (Table 2). Two cases were assessed using different pathological materials from the same individual. Case #29, positive for *ROS1* rearrangement via FISH on a pleural biopsy collected in 2017, was also tested on a cell block obtained from a pleural effusion. The Idylla results were concordant, despite the five Cqs of difference in the RNA control (case 29-B) (Table 2). This individual in 2021 had a disease relapse. A new pleural biopsy was performed, which, when analyzed with Idylla, again confirmed a *ROS1* rearrangement (case #29-C). The reanalysis of the 2017 and 2021 pleural biopsies by the NGS Oncomine Dx panel identified an SDC4(2) -ROS1(32) fusion (Table 2). Case #33 was *RET*-positive, as identified via RT-PCR with the AmoyDx^®^ PLC Panel (AmoyDx). The Idylla assay performed on both a biopsy and a concomitant cell block was found to be concordant with the previous analysis. NGS subsequently confirmed a specific KIF5B (16)-RET (12) rearrangement. Overall, 14 of the 17 cases analyzed with Idylla were in agreement with the relative reference test. To verify the reproducibility and robustness of the assay in different pre-analytical conditions, cases #29 (*ROS1*), #33 (*RET*), #36 (*MET* ex14 skipping) and #38 (*NTRK/3*) were reanalyzed from sections prepared and stored at RT for 60, 30, 49 and 36 days, respectively. The results were in agreement with the first molecular determination (cases #29, 33, 36, 38 and, respectively, #29-B, 33-B, 36-B, 38-B, Table 2).

Finally, the mean age of blocks at testing was 24.2 months with ALK testing and 21.6 months with the other gene fusions. Considering an arbitrary cut-off of 1 year (12 months), the median Cq in the subgroup of fewer than 12 months were RNA = 27.7 and DNA = 29.0, while Cqs of RNA = 28.6 and DNA = 29.6 were observed in the subgroup ≥ 12 months.

## 4. Discussion

In this study, we evaluated the performance of the new Idylla^TM^ GeneFusion assay for simultaneous assessment of the *ALK*, *ROS1*, *RET* and *NTRK*s gene translocations and *MET* ex14 skipping mutations. For each of these gene alterations, effective TKI-based therapy is available. The test is fully automated and involves the execution of a single step consisting of the introduction of a scraped tissue sample into a cartridge that incorporates all the necessary internal controls with a run time of up to 180 min. The evaluated cohort consisted of thirty-five lung adenocarcinomas, one neuroendocrine lung cancer, one combined lung carcinoma and two (*NTRK/3*-positive) secretory parotid gland carcinomas. Although 17 of the 39 cases used for the study were related to pathological materials older than 2 years (range 2–10 years), Idylla invalid runs were not observed.

In the first part of the study, we evaluated 22 cases previously analyzed for *ALK* alterations, of which 19 scored positive via IHC (ALK-positive), two exhibited dubious ALK IHC staining and one was *ALK*-positive through RT-PCR, as detected using Amoy-Dx technology (PLC panel) (Table 1). All were concordant, except the two with uncertain IHC interpretations. Both of the doubtful cases were shown to be negative through both the Idylla and FISH approaches. So, all the frankly positive ALK-positive cases were in agreement. Case #5 was retested 21 days after the first Idylla evaluation to probe its feasibility on sections previously prepared and stored in the dark at RT. Repeated testing confirmed the previous result (Table 1). In the second part of the study, we analyzed 17 cases related to samples positive for *ROS1*, *RET* and *NTRK*s and *MET* ex14 skipping previously analyzed through FISH, RT-PCR, IHC or NGS. Seven of the nine *ROS1*-positive cases identified through FISH or IHC were correctly detected (Table 2). Unfortunately, the residual pathological tissue from the two Idylla-negative cases was not sufficient for further NGS evaluation. 

The Idylla^TM^ GeneFusion assay qualitatively detects specific gene fusions for *ALK*, *ROS1* and *RET* as well as their corresponding 3′/5′ expression imbalance (3′/5′EI). Only 3′/5′EI was detected for *NTRK1/2/3,* while the specific exon 14 skipping alteration was identified for *MET*. Additionally, 3′/5′EI assays have the advantage that fusion transcripts with uncommon breakpoints or unknown fusion partners may still be identified, providing an indication of the presence of an uncommon fusion event. In our study, out of twenty *ALK* assessments, we detected twenty *ALK*-positive cases, eighteen of which exhibited an *ALK*-specific fusion along with a positive 3′/5′EI, while only two cases (#3 and #13) showed the 3′/5′EI with no specific *ALK* fusion detection. Since these cases had a positive ALK IHC, we can speculate that an *ALK* translocation with a fusion partner not included in the Idylla assay design was present. 

Similarly, 3′/5′ EI along with specific fusion was seen for the three *RET*-positive cases analyzed (Table 2). Our findings with respect to *ROS1* fusion detection were different. Of the seven fusion-specific cases, none showed a *ROS1* 3′/5′EI, suggesting that uncommon *ROS1* fusions, not present in the Idylla design, may be missed (Table 2). This could be explained by a biological phenomenon related to the variability of gene fusion expression depending on the gene and the type of fusion product that can be further affected by low tumor content. Recently, it has been reported that, differently from *ALK* and *RET*, high wild-type *ROS1* endogenous expression in normal tissue may obscure *ROS1* 3′/5′ EI detection in the tumor counterpart [18]. Our findings are in agreement with these observations and further suggest that, for biological reasons, 3′/5′EI-based *ROS1* gene fusion detection via Idylla and RT-PCR in general may be less than efficient. A major limitation of the present study lies in the scarcity of NTRK-positive tested specimens, in fact, only two NTRK/3 rearranged samples from two salivary gland carcinomas were available. Given the very low NTRK fusion frequency in NSCLC, ranging from about 0.07–3.3% for NTRK1, 0.02–0.2% for NTRK2 and 0.08% for NTRK3, a specific large multi-institutional study would be needed to fully validate the Idylla platform for this biomarker in NSCLC [19].

A *MET* ex14 skipping sample shown to be positive via NGS using the Oncomine Dx panel was not confirmed. A subsequent analysis with a second RT-PCR assay, EasyPGX^®^, was in agreement with Idylla, raising the suspicion of an NGS false positive result. An alert has recently been reported showing that false *MET* ex14 skipping might be caused by the homopolymeric error of the splice donor site with the Oncomine Dx Target test [20,21]. These false positives could be distinguished by relatively low read counts. The discordant case was in fact characterized by low read counts (275 mutated reads/960344 total mapped reads). Moreover, this case was also analyzed via EasyPGX^®^ ready ALK, ROS1, RET, MET (Diatech Pharmacogenetics) and resulted in a negative. In the third part of the study, we challenged the test to evaluate robustness and reproducibility using different pathological specimens from the same selected individuals or performing the assay under different pre-analytical conditions. In eleven cases, two, three or four repeat tests were performed. For cases #27 and #28 (*ROS1*-positive), a second evaluation was carried out on a more limited region of the tumor area (Figure 2). For other selected cases, the evaluation was carried out on alternative pathological materials from the same individual, such as a lymph node, pleural biopsy or cell block. Finally, five cases were repeated on sections previously prepared and stored at RT for up to 60 days. All repetitions of the Idylla assay confirmed the previous result (Table 1 and Table 2). So, RNA of sufficient quality for evaluation with the Idylla GeneFusion can be obtained under different pre-analytical conditions.

Past intensive research has taught us that RNA degradation and fragmentation can be caused by various factors such as cellular autolysis, tissue necrosis, cold ischemia and formalin fixation. Furthermore, environmental RNases can seriously endanger the integrity of the RNA after the extraction. Therefore, it is recommended to store the RNA at −20 °C or, even better, at −80 °C, keep it on ice during the handling phases and avoid repeated freezing and thawing. The robustness of the Idylla assay may lie in its specific design, comporting that the extraction of nucleic acids and subsequent reverse transcription and RT-PCR reactions take place sequentially in a closed cartridge, i.e., in an RNase-free environment. This allows the avoidance of manipulations that can be deleterious for nucleic acid integrity. A prototype of the Idylla GeneFusion assay has been recently evaluated in a multicenter study, showing a concordance with reference methods of 89% [22]. Three papers have been published using the definitive, commercially available Idylla RUO assay. All three compared Idylla to NGS as the gold standard. In the first, the presence of *NTRK* gene fusion in a pan-cancer setting comparing Idylla versus the RNA-based Oncomine Focus Assay (Thermo Fisher Scientific) was evaluated. The concordance was 92.7%, showing a clear potential of Idylla in identifying *NTRK* fusions [23]. In the second, the presence of gene fusions and oncogenic mutations in MLH1-deficient and *BRAF* V600E wild-type colorectal cancers were assessed. The concordance between the Idylla^TM^ GeneFusion assay and a novel FusionPlex Lung v2 RNA-based NGS panel test (Thermo Fisher Scientific) for *NTRK1*, *ALK* and *RET* were, respectively, 100%, 88.1% and 94.9% [24]. In the third, 143 cancer cases, including 108 NSCLCs, previously tested using a clinically validated NGS assay (MSK-IMPACT) were reanalyzed with Idylla. Testing was successful in 142 (99%) with a 97% agreement [25]. Up to ten-year-old paraffin-embedded blocks were used in the studies, demonstrating that RNA of sufficient quality for evaluation with Idylla^TM^ GeneFusion can be obtained from long-term archived material. Unlike the aforementioned studies, NGS was not our gold-standard reference method. Indeed, in our cohort, 31/39 cases (77%) (79.4%) were evaluated via IHC or FISH. A premise must be made: RNA-based NGS methods are strongly influenced by the fragmentation of nucleic acids extracted from FFPE samples, which can lead to a lower reading coverage due to the small size of the RNA templates [26]. Cases that cannot be assessed via NGS are excluded and must be analyzed differently. Consequently, when NGS is the reference, one can argue that the experimental conditions of the assay comparison can be defined as “optimal”. On the other hand, when IHC or FISH are used as the reference methods, the quality of the RNA is unknown, as these “in situ” methods are not RNA-dependent, as also recently demonstrated by Ambrosini-Spaltro et al. [27]. So, in our opinion, having used different reference methods is not a limitation, but instead adds some practical observations to previous studies, particularly when considering small samples that fail with or result in inadequate NGS.

In line with the aforementioned reports, we used old FFPE blocks, of which 17/39 were more than two years old (on a ten-year-old block, we carried out a double *ALK* assessment on a surgical specimen and concomitant lymph node metastasis), and, in addition, for the first time, we demonstrated that sections prepared and stored for several days may still be valid for determination with Idylla. So, tumor sections from NSCLC patients who have been only partially profiled and need additional information for their therapeutic treatment may be safely shipped to a referral molecular laboratory.

Of note, Boppudi et al. [28] recently investigated Idylla assays, including the Gene-Fusion Assay, in several tissues and source materials and in archival tissue dating back 20 years. The authors evidenced a 100% specificity, 96.3% sensitivity for specific gene fusion and 80% sensitivity for expression imbalance, while the low quality of the RNA extracted from formalin-fixed paraffin-embedded blocks and hematoxylin-eosin-stained slides failed to demonstrate a specific gene fusion or expression imbalance.

**Figure 3:**
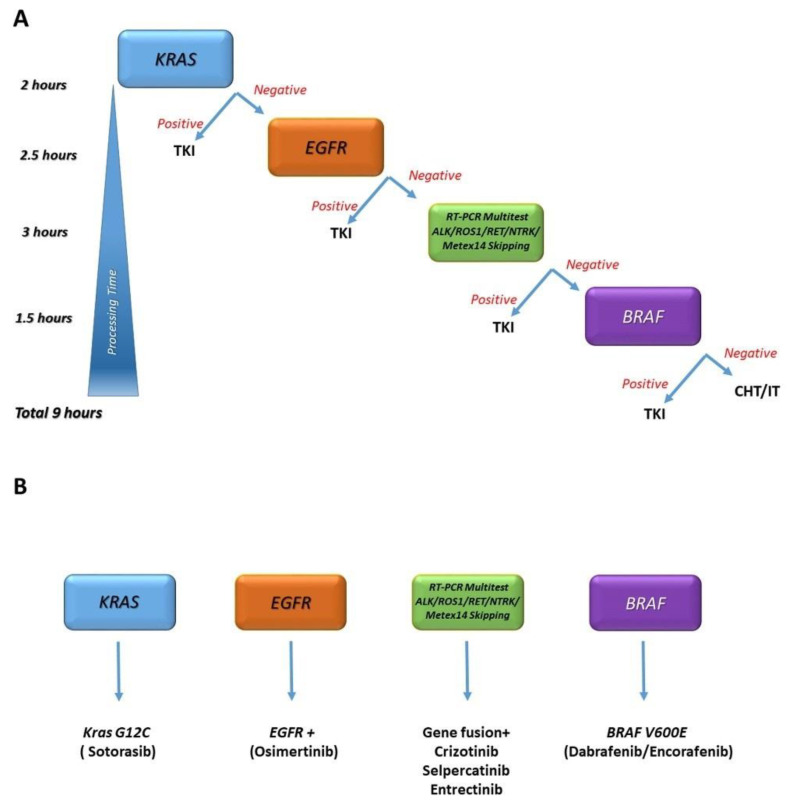
One day NSCLC profiling with the Idylla platform. Idylla is a fully integrated, cartridge-based assay that provides automated sample processing, including deparaffinization, tissue digestion, nucleic acid extraction, reverse transcription of mRNA, real-time PCR amplification and detection of the targeted sequences. Single *KRAS*, *EGFR* and *BRAF* assays are available, while the GeneFusion assay is a multitest able to simultaneously detect *ALK*, *ROS1*, *RET* and *NTRK* translocations, along with *MET* ex14 skipping mutations. Using a single module, one test at a time can be performed, and testing can be stopped if a *KRAS*, *EGFR* or *BRAF* mutation is found, according to the algorithm (cheaper solution, panel (**A**)). When scaling up using more modules, the profiling can be more time-efficient but also more expensive (panel (**B**)).

In conclusion, we demonstrated that multiple disease-defining translocations can be robustly identified with a single low-cost assay within a short time (3 h) after the reporting of results. The set of Idylla assays also includes *EGFR*, *KRAS* and *BRAF*. These oncogenic drivers, including the gene fusions detailed in this work, are believed to be mutually exclusive [29]. The combination of these events makes realistic the possibility of a same-day, complete, advanced NSCLC patient molecular assessment and allows the proposition of an algorithm to be applied to individuals in need of prompt acquisition of molecular information for therapy initiation (Figure 3).

## 5. Conclusions

Updated international guidelines suggest that NGS should be the preferred procedure for NSCLC sample evaluation for predictive biomarkers, but NGS facilities are not available everywhere. Alternative molecular techniques are rapidly evolving, each with different characteristics and performance in terms of turn-around time, sensitivity, specificity and required personnel skills. Among these, we suggest Idylla as a valid stand-alone test for laboratories lacking NGS facilities or representing a viable alternative option for ultra-rapid NSCLC patient profiling when time-efficient NGS evaluation is not possible.

## Figures and Tables

**Table 1:** Patient characteristics and Idylla^TM^ GeneFusion performance versus reference method for ALK cohort.

Sample ID	Gender	Age	Histology	Block Age at Testing (Months)	Specimen	Tumor Cell Content	Reference Method	*ALK* Specific Fusion	*ALK* 3′5′ Imbalance	Idylla Overall Result	Comparison to Reference	Cq RNA Controls	Cq DNA Controls
#1 #1-B	M	47	ADC ADC	3 2	biopsy re-biopsy	>50% >50%	IHC pos IHC pos	Y Y	Y Y	pos pos	C C	26.6 27.0	27.6 29.4
#2	F	65	ADC	1	biopsy	>50%	IHC pos	Y	Y	pos	C	29	29.9
#3	F	87	ADC	1	biopsy	>50%	IHC pos	N	Y	pos	C	27.7	28.5
#4	M	75	LCNEC	13	surgical	>50%	IHC dubious	N	N	*neg*	D	26	26.2
#5	F	66	ADC	26	biopsy	>50%	IHC pos	Y	Y	pos	C	27.8	29.8
#5-B					after 3 weeks	>50%	Not performed	Y	Y	pos		28	29.8
#6	M	54	ADC	25	pleural biopsy	>50%	IHC pos	Y	Y	pos	C	26.3	28.9
#7	F	77	ADC	31	biopsy	>50%	IHC pos	Y	Y	pos	C	30.5	31.6
#8	F	48	ADC	24	biopsy	>50%	IHC pos	Y	Y	pos	C	30.1	31.3
#9	F	52	ADC	29	biopsy	>50%	IHC pos	Y	Y	pos	C	29.1	31.3
#10	M	69	ADC	12	biopsy	>50%	IHC pos	Y	Y	pos	C	27.5	30.4
#11	M	64	ADC	17	pleural biopsy	>50%	IHC pos	Y	Y	pos	C	27	29.9
#12	M	41	ADC	27	pleural biopsy	>50%	IHC pos	Y	Y	pos	C	29.3	31.8
#13	M	60	ADC	19	surgical	>50%	IHC pos	N	Y	pos	C	27.3	28.5
#14	M	72	ADC+SCLC	1	biopsy	>50%	IHC dubious	N	N	*neg*	D	26.3	27.1
#15	M	52	ADC	3	surgical	>50%	IHC pos	Y	Y	pos	C	25.3	28.5
#15-B				4	surgical (metastatic lymph node)	>50%	Not performed	Y	Y	pos		28.4	30
#16	M	44	ADC	15	surgical	>50%	IHC pos	Y	Y	pos	C	27.2	28.7
#16-B				15	surgical (metastatic lymph node)	>50%	Not performed	Y	Y	pos		27.3	29
#17	F	69	ADC	123	surgical	>50%	FISH pos	Y	Y	pos	C	29.6	27.2
#17-B				123	surgical (metastatic lymph node)	>50%	*Not performed*	Y	Y	pos		28.9	28.9
#17-C			ADC	1	re-biopsy	>50%	IHC pos	Y	Y	pos	C	28.6	31.2
#18	M	71	ADC	1	biopsy	>30%	IHC pos	Y	Y	pos	C	28	28.2
#18-B				1	pleural biopsy	>50%	Not performed	Y	Y	pos		27.5	28.1
#19	F	85	ADC	3	biopsy	50%	Amoy-DX pos	Y	Y	pos	C	27	27.7
#20	M	57	ADC	13	biopsy	>50%	IHC pos	Y	Y	pos	C	29	31.3
#21	F	38	ADC	84	biopsy	>50%	IHC pos	Y	Y	pos	C	27.3	28.5
#22	M	51	ADC	85	biopsy	>50%	IHC pos	Y	Y	pos	C	28.4	29.7

Abbreviations: M: male; F: female; ADC: adenocarcinoma; LCNEC: large cell neuroendocrine carcinoma; SCLC: small cell lung carcinoma; IHC: immunohistochemistry; FISH: fluorescent in situ hybridization; pos: positive; neg: negative; N/A: not applicable; Cq: cycle quantification; C: concordant; D: discordant.

**Table 2:** Patient characteristics and Idylla GeneFusion performance versus reference method for *ROS1/RET/MET/NTRK* cohort.

Sample ID	Gender	Age	Histology	Block Age at Testing (months)	Specimen	Tumor Cell Content	Reference Method/Gene Target	Gene-Specific Fusion	Gene 3′-5′ Imbalance	Idylla Overall Result	Other Method	Comparison to Reference	Cq RNA Controls	Cq DNA Controls
#23	F	80	ADC	28	pleural biopsy	>50%	FISH/ROS1 Pos	Y	N	Y	None	C	30.4	31.5
#24	F	63	ADC	2	cell block	30%	FISH/ROS1 Pos	Y	N	Y	None	C	29.8	30.9
#25	F	47	ADC	20	biopsy	>50%	FISH/ROS1 Pos	N	N	N	None	D	29.2	29.8
#26	M	77	ADC	19	biopsy	>50%	FISH/ROS1 Pos	N	N	N	None	D	30.2	31.1
#27	F	72	ADC	44	surgical	>50%	FISH/ROS1 Pos	Y	N	Y	None	C	27.6	27.2
#27-B					small md			Y	N	Y	None	C	30	29.5
#28	F	75	ADC	74	surgical	>50%	FISH/ROS1 Pos	Y	N	Y	None	C	28.8	28.3
#28-B					small md			Y	N	Y	None	C	30.3	30.2
#29	F	49	ADC	48	pleural biopsy	>50%	FISH/ROS1 Pos	Y	N	Y	NGS/SDC4(2)-ROS1(32)	C	28.2	30.9
#29-B				48	cell block	30%		Y	N	Y	None		33	31.4
#29-C				1	pleural biopsy	>50%		Y	N	Y	NGS/SDC4(2)-ROS1(32)		27.1	30.1
#29-D					after 60 days			Y	N	Y	None		27.5	29.7
#30	M	74	ADC	1	biopsy	>50%	IHC/ROS1 Pos	Y	N	Y	None	C	31.2	33.5
#31	F	71	ADC	4	biopsy	20%	AmoyDx/RET Pos	Y	Y	Y	None	C	29	30
#32	M	49	ADC	3	biopsy	>50%	AmoyDx/RET Pos	Y	Y	Y	None	C	28	27.8
#33	F	75	ADC	3	biopsy	40%	AmoyDx/RET Pos	Y	Y	Y	NGS/KIF5B(16)-RET(12)	C	27.5	27.1
#33-B				1	cell block	20%		Y	Y	Y	None		28.1	27.7
#33-C					after 30days			Y	Y	Y	None		29.4	29.8
#34	M	69	ADC	23	surgical	>50%	NGS/MET Pos	N	N	N	EasyPGX/Negative	D	26.6	27.8
#35	F	81	ADC	11	surgical	>50%	EasyPgx/MET Pos	Y	N/A	Y	None	C	26.1	26.6
#36	F	71	ADC	7	surgical	>50%	EasyPgx/MET Pos	Y	N/A	Y	None	C	27.3	27.8
#36-B					after 49days			Y	N/A	Y	None	C	27.9	27.1
#37	M	31	PGS-carcinoma	14	surgical	>50	NGS/ETV6(5) - NTRK3(15)	N/A	NTRK/3	Y	None	C	28.5	28.7
#38	M	57	PGS- carcinoma	46	surgical	>50%	IHC PanNTRK/NTRK Pos	N/A	NTRK/3	Y	None	C	28.1	28.9
#38-B					after 36 days			N/A	NTRK/3	Y	None	C	30.5	29.6
#39	M	55	ADC	35	pleural biopsy	>50%	IHC/ROS1 Pos	Y	N	Y	FISH/ROS1	C	30.2	31.9

Abbreviations: M: male; F: female; ADC: adenocarcinoma; PG: parotid gland secretory; IHC: immunohistochemistry; FISH: fluorescent in situ hybridization; md: microdissection; Pos: positive; Neg: negative; N/A: not applicable; Sample collect: sample collection date; Cq: cycle quantification; Y: yes; N: no; C: concordant; D: discordant.

## Data Availability

All data are available upon reasonable request.

## References

[1] Kris M.G., Johnson B.E., Berry L.D., Kwiatkowski D.J., Iafrate A.J., Wistuba I.I., Varella-Garcia M., Franklin W.A., Aronson S.L., Su P.F. (2014). Using multiplexed assays of oncogenic drivers in lung cancers to select targeted drugs. JAMA.

[2] Lee S.E., Lee B., Hong M., Song J.Y., Jung K., Lira M.E., Mao M., Han J., Kim J., Choi Y.L. (2015). Comprehensive analysis of RET and ROS1 rearrangement in lung adenocarcinoma. Mod. Pathol..

[3] Pan Y., Zhang Y., Li Y., Hu H., Wang L., Li H., Wang R., Ye T., Luo X., Zhang Y. (2014). ALK, ROS1 and RET fusions in 1139 lung adenocarcinomas: A comprehensive study of common and fusion pattern-specific clinicopathologic, histologic and cytologic features. Lung Cancer.

[4] Howlader N., Forjaz G., Mooradian M.J., Meza R., Kong C.Y., Cronin K.A., Mariotto A.B., Lowy D.R., Feuer E.J. (2020). The Effect of Advances in Lung-Cancer Treatment on Population Mortality. N. Engl. J. Med..

[5] Jordan E.J., Kim H.R., Arcila M.E., Barron D., Chakravarty D., Gao J., Chang M.T., Ni A., Kundra R., Jonsson P. (2017). Prospective Comprehensive Molecular Characterization of Lung Adenocarcinomas for Efficient Patient Matching to Approved and Emerging Therapies. Cancer Discov..

[6] Nozaki Y., Yamamoto H., Iwasaki T., Sato M., Jiromaru R., Hongo T., Yasumatsu R., Oda Y. (2020). Clinicopathological features and immunohistochemical utility of NTRK-, ALK-, and ROS1-rearranged papillary thyroid carcinomas and anaplastic thyroid carcinomas. Hum. Pathol..

[7] Solomon J.P., Hechtman J.F. (2019). Detection of NTRK Fusions: Merits and Limitations of Current Diagnostic Platforms. Cancer Res..

[8] Yang S.R., Aypar U., Rosen E.Y., Mata D.A., Benayed R., Mullaney K., Jayakumaran G., Zhang Y., Frosina D., Drilon A. (2021). A Performance Comparison of Commonly Used Assays to Detect RET Fusions. Clin. Cancer Res..

[9] Park G., Kim T.H., Lee H.O., Lim J.A., Won J.K., Min H.S., Lee K.E., Park D.J., Park Y.J., Park W.Y. (2015). Standard immunohistochemistry efficiently screens for anaplastic lymphoma kinase rearrangements in differentiated thyroid cancer. Endocr. Relat. Cancer.

[10] (2015). Memorial Sloan Kettering Cancer Center, West Harrison, N.Y. Mod. Healthc..

[11] Mosele F., Remon J., Mateo J., Westphalen C.B., Barlesi F., Lolkema M.P., Normanno N., Scarpa A., Robson M., Meric-Bernstam F. (2020). Recommendations for the use of next-generation sequencing (NGS) for patients with metastatic cancers: A report from the ESMO Precision Medicine Working Group. Ann. Oncol..

[12] Srivastava R. (2022). Applications of artificial intelligence multiomics in precision oncology. J. Cancer Res. Clin. Oncol..

[13] Williams H.L., Walsh K., Diamond A., Oniscu A., Deans Z.C. (2018). Validation of the Oncomine() focus panel for next-generation sequencing of clinical tumour samples. Virchows Arch..

[14] Aguado C., Gimenez-Capitan A., Roman R., Rodriguez S., Jordana-Ariza N., Aguilar A., Cabrera-Galvez C., Rivas-Corredor C., Lianes P., Viteri S. (2020). RNA-Based Multiplexing Assay for Routine Testing of Fusion and Splicing Variants in Cytological Samples of NSCLC Patients. Diagnostics.

[15] Evangelista A.F., Zanon M.F., Carloni A.C., de Paula F.E., Morini M.A., Ferreira-Neto M., Soares I.C., Miziara J.E., de Marchi P., Scapulatempo-Neto C. (2017). Detection of ALK fusion transcripts in FFPE lung cancer samples by NanoString technology. BMC Pulm. Med..

[16] Lira M.E., Choi Y.L., Lim S.M., Deng S., Huang D., Ozeck M., Han J., Jeong J.Y., Shim H.S., Cho B.C. (2014). A single-tube multiplexed assay for detecting ALK, ROS1, and RET fusions in lung cancer. J. Mol. Diagn..

[17] WHO Classification of Tumours Editorial Board (2021). Thoracic Tumours.

[18] Lira M.E., Kim T.M., Huang D., Deng S., Koh Y., Jang B., Go H., Lee S.H., Chung D.H., Kim W.H. (2013). Multiplexed gene expression and fusion transcript analysis to detect ALK fusions in lung cancer. J. Mol. Diagn..

[19] Liu F., Wei Y., Zhang H., Jiang J., Zhang P., Chu Q. (2022). NTRK Fusion in Non-Small Cell Lung Cancer: Diagnosis, Therapy, and TRK Inhibitor Resistance. Front. Oncol..

[20] Teishikata T., Shiraishi K., Shinno Y., Kobayashi Y., Kashima J., Ishiyama T., Yoshida T., Mori T., Yatabe Y. (2021). An Alert to Possible False Positives With a Commercial Assay for MET Exon 14 Skipping. J. Thorac. Oncol..

[21] Subramanian J., Tawfik O. (2021). Detection of MET exon 14 skipping mutations in non-small cell lung cancer: Overview and community perspective. Expert. Rev. Anticancer Ther..

[22] Depoilly T., Garinet S., van Kempen L.C., Schuuring E., Clave S., Bellosillo B., Ercolani C., Buglioni S., Siemanowski J., Merkelbach-Bruse S. (2021). Multicenter Evaluation of the Idylla GeneFusion in Non-Small-Cell Lung Cancer. J. Mol. Diagn..

[23] Sorber L., Van Dorst B., Bellon E., Zwaenepoel K., Lambin S., De Winne K., Lardon F., Pauwels P., Siozopoulou V. (2022). NTRK Gene Fusion Detection in a Pan-Cancer Setting Using the Idylla GeneFusion Assay. J. Mol. Diagn..

[24] Ukkola I., Nummela P., Kero M., Tammio H., Tuominen J., Kairisto V., Kallajoki M., Haglund C., Peltomaki P., Kytola S. (2022). Gene fusions and oncogenic mutations in MLH1 deficient and BRAFV600E wild-type colorectal cancers. Virchows Arch..

[25] Chu Y.H., Barbee J., Yang S.R., Chang J.C., Liang P., Mullaney K., Chan R., Salazar P., Benayed R., Offin M. (2022). Clinical Utility and Performance of an Ultrarapid Multiplex RNA-Based Assay for Detection of ALK, ROS1, RET, and NTRK1/2/3 Rearrangements and MET Exon 14 Skipping Alterations. J. Mol. Diagn..

[26] Fujii T., Uchiyama T., Matsuoka M., Myojin T., Sugimoto S., Nitta Y., Okabe F., Sugimoto A., Sekita-Hatakeyama Y., Morita K. (2019). Evaluation of DNA and RNA quality from archival formalin-fixed paraffin-embedded tissue for next-generation sequencing - Retrospective study in Japanese single institution. Pathol. Int..

[27] Ambrosini-Spaltro A., Farnedi A., Calistri D., Rengucci C., Prisinzano G., Chiadini E., Capelli L., Angeli D., Bennati C., Valli M. (2022). The role of next-generation sequencing in detecting gene fusions with known and unknown partners: A single-center experience with methodologies’ integration. Hum. Pathol..

[28] Boppudi S., Scheil-Bertram S., Faust E., Annamneedi A., Fisseler-Eckoff A. (2022). Assessing and Evaluating the Scope and Constraints of Idylla Molecular Assays by Using Different Source Materials in Routine Diagnostic Settings. Int. J. Mol. Sci..

[29] De Marchi F., Haley L., Fryer H., Ibrahim J., Beierl K., Zheng G., Gocke C.D., Eshleman J.R., Belchis D., Illei P. (2019). Clinical Validation of Coexisting Activating Mutations Within EGFR, Mitogen-Activated Protein Kinase, and Phosphatidylinositol 3-Kinase Pathways in Lung Cancers. Arch. Pathol. Lab. Med..

